# Clinical study of modified photodynamic therapy combined with Taohong Siwu Decoction in treating hypertrophic scar after severe burn

**DOI:** 10.1016/j.clinsp.2023.100295

**Published:** 2023-10-30

**Authors:** Zhenguo Zhang, Shidong He, Qiaoxia Yu, Jiayue Ding

**Affiliations:** a Plastic & Reconstruct Surgery, Lishui People's Hospital, Zhejiang, China; b Department of Surgery, Yunhe People's Hospital, Yunhe, Zhejiang, China; c Chongtou Central Health Center, Yunhe, Zhejiang, China

**Keywords:** Severe Burn, Hypertrophic Scar, Modified Photodynamic Therapy, Taohong Siwu Decoction, Clinical Efficacy, Satisfaction, Security

## Abstract

**Objective:**

The aim of this study was to study the curative effect and the relative mechanism of modified photodynamic therapy combined with Taohong Siwu Decoction in the treatment of hyperplastic scar after severe burn, in order to provide a stable, safe and satisfactory scheme for scar repair.

**Methods:**

Forty cases with hyperplastic scars after severe burns admitted to the plastic surgery department from May 2021 to May 2022 were divided into a control group and an observation group by means of the random number table method. The control group was treated with ordinary laser therapy combined with Taohong Siwu Decoction, while the observation group was treated with modified photodynamic therapy combined with Taohong Siwu Decoction. The Vancouver Scar Scale (VSS) was assessed in both groups, and the clinical effectiveness of both groups was compared. HE-staining was performed on the scar tissue of the same patient before and after treatment to observe the changes in the arrangement of fibroblasts. The Vascular Endothelial Growth Factor (VEGF), β-Transforming Growth Factor (TGF-β), and Platelet-Derived Growth Factor (PDGF) in the tissue samples of both groups were detected by quantitative real-time PCR. The patients were followed up for 6 months, and their satisfaction, side effects, and scar recurrence were observed.

**Results:**

Compared with the control group, the VSS score in the observation group was lower (p < 0.05). The therapeutic effect of the observation group was superior to the control group after 3 months (p < 0.05). After 3-months of therapy, the arrangement of fibroblasts in the scar became looser in two groups, and the observation group was more looser. The VEGF, TGF-β and PDGF levels in tissue samples of the observation group were lower than those in the control group after 3 months of treatment (p < 0.05). The satisfaction of the observation group was higher than that of the control group (p < 0.05). The adverse reactions between the two groups showed no difference (p > 0.05), while the recurrence rate was lower in the observation group (p < 0.05).

**Conclusion:**

Modified photodynamic therapy combined with Taohong Siwu Decoction shows remarkable efficacy in patients with hyperplastic scars after severe burns. It can improve the color, thickness, vascular distribution, and softness of the scar, and reduce the level of cytokines related to tissue repair. At the same time, it can improve patients' satisfaction with the aesthetic appearance and reduce the recurrence rate, providing a new comprehensive therapy that is safer and more effective, simple and quick, and easy to promote in the clinic.

## Highlights

• MPT combined with THSD shows remarkable efficacy in hyperplastic scar patients.
• MPT combined with THSD can reduce the level of cytokines related to tissue repair.
• The study provides a new comprehensive therapy for hyperplastic scar patients.

## Introduction

Severe burn is very common clinically, because it can involve the deep dermis, resulting in the incidence of post-healing scars as high a 83%, and hypertrophic scars are the main scar, which is difficult to recover, seriously affecting the physical and mental health of patients.^1^ Hypertrophic scars are pathologically characterized by excessive accumulation of extracellular matrix such as collagen I and fibroblasts, which can continue to grow and invade adjacent healthy skin in the case of keloids. Hypertrophic scars not only affect the aesthetic appearance of patients, they can also grow across joints or cause joint contracture, which may lead to serious dysfunction.^2^ The treatment of hypertrophic scars has always been one of the major challenges in plastic surgery. Although many therapeutic methods have been explored after years of research, including drug therapy, physical therapy, surgical therapy, laser therapy and comprehensive therapy (pressure therapy, scar massage, scar stretching, intra-scar drug injection, etc.),^3^ there is still no satisfactory treatment plan.

Photodynamic Therapy (PDT) is a type of laser therapy, because of the advantage of high selectivity and few side effects, which has been used to treat hypertrophic scar.^4^ Among photosensitivities, 5-Aminolevulpionic Acid (ALA) has been shown to be an excellent candidate for treatment of topical dermatological without significant side effects. However, its poor penetration into hyperplastic scar tissue and fibroblasts and low quantum yield of cytotoxic Reactive Oxygen Species (ROS) make it less effective.^5^ Thus, a lot of attention in focused on improving the permeability of ALA and the quantum yield of ROS in treating hypertrophic scar by photodynamic therapy. In previous studies, the authors developed ALA/gold Nanoparticles (AuNP) loaded nanoethosomes (ES) (A/A-ES) with good biocompatibility and penetration. In addition, it was shown that A/A-ES showed strong absorbance in the 600‒650 nm range due to plasma coupling between adjacent AuNPs, which allowed the stimulation of A/A-ES with He-Ne laser to generate both heat and ROS, thus promoting hypertrophic scar fibroblasts cell apoptosis.^6^ The healing process of hyperplastic scar is a complex process involving multiple mechanisms, including inflammatory response, coagulation, granulation tissue formation, and regeneration of epithelial tissues.^7^ Taohong Siwu Decoction was originally in Yizong Jinjian, which is an ancient traditional Chinese medicine book written in the Qing Dynasty, and widely used in improving blood circulation and alleviating blood stagnation. Several studies have shown that Taohong Siwu Decoction can effectively inhibit the inflammatory reaction of skin scar tissue and promote the repair of scars.^8^^,^^9^

Although previous studies have shown that A/A-ES is a promising topical ALA and AuNP transdermal drug delivery system with great potential for application in photodynamic therapy for hypertrophic scarring, its safety, and therapeutic effects on patients with severe post-burn scarring need further discussion. Therefore, the purpose of this study is to investigate the therapeutic effect and mechanism of modified photodynamic therapy combined with Taohong Siwu Decoction on hyperplastic scar after severe burn.

## Research methods

### *Ethical approval of research protocol*

Ethical approval was obtained from the hospital review board for theis prospective, randomized, controlled trial. The ethical approval number is 2022‒190. Written informed consent was obtained from patients or their guardians prior to participation in this study. Both patients and researchers were blinded.

### *Patients*

Patients with hypertrophic scars from May 2021 to May 2022 were enrolled in this study. Inclusion criteria: age range 18∼60 years old; the ethnicity belongs to Han nationality; the causation of scar was severe burn; all scar sites were facial neck; scar thickened and raised irregular shape. Exclusion criteria: active skin diseases such as herpes existed in the treatment site; recent use of photosensitive drugs; obvious cicatricial constitution; pregnant or lactating women; heart, liver, and kidney dysfunction. According to the above criteria, a total number of 40 cases were enrolled and randomly assigned to the control group and an observation group, with 20 patients in each group.

### *Preparation of A/A-ES*

AuNPs were synthesized and loaded into liposomes (ES) using ultrasonication. Biocompatible A/A-ES suspensions were obtained by loading ALA into ES at 20% encapsulation rate using the pH gradient method.

### *Treatment method*

The control group received ordinary laser therapy combined with Taohong Siwu Decoction, while the observation group obtained modified photodynamic therapy combined with Taohong Siwu Decoction. Specific methods: Both groups were treated with KL-type fractional laser therapy. After routine facial cleaning, 5% lidocaine ointment was applied to the scar as well as the normal skin around the scar for one hour, and more than the ointment was wiped off with saline. The control group received normal laser mode, while the study group received CO_2_ fractional laser fractional mode treatment (energy: 80 mj, 20% coverage) on the surface of the scar. First, 0.2% mass-to-volume A/A-ES suspension was applied to the surface of the scar, covered with cling film, fixed, and sealed for 3h. After sealing, the excess A/A-ES suspension was wiped off the surrounding area, and the non-scarred area was covered with tinfoil, then irradiated with a red light at a wavelength of 633 nm for 20 min at an energy density of 90 mw.cm^−2^. After the irradiation, the patient was instructed to apply a cold compress for 20 minutes. The treatment was performed once a month for a total of 3 times. All patients were treated with laser in combination with Taohong Siwu Decoction, which consists of Hong-Hua (20g), Tao-Ren (20g), Lian-Qiao (20g), Xia-Ku-Cao (20g), Mu-Li (20g), Zhao-Jiao-Ci (20g), Fa-Ban-Xia (20g), Fu-Ling (15g), Chi-Shao (15g), Dang-Gui (15g), Chuan-Xiong (15g), Chen-Pi (12g), Zhe-Bei-Mu (12g), Shu-Di-Huang (12g), Sheng-Gan-Cao (5g), Jin-Yin-Hua (5g). The above drugs were decocted with water, one dose a day for 3 months.

### *Outcome measures*

The Vancouver Scar Scale (VSS) scores were evaluated and used to assess the clinical efficacy of both groups. The VSS was evaluated from color, blood vessel distribution, thickness, and softness with a total score of 15 points. Score decline rate = (VSS score before treatment - VSS score after treatment)/VSS score before treatment ×100%, score decline rate ≥ 90% are cured; 60% ≤ score decline rate < 90% is effective; 20% ≤ score decline rate < 60% is improved; score decline rate < 25% is ineffective. Clinical response rate = (cured cases + effective cases + improved cases) / total cases ×100%. The arrangement of fibroblasts in scar tissue was obtained by HE-staining. The VEGF, TGF-β, and PDGF in the tissue samples of both groups were measured by enzyme-linked immunosorbent assay, and immunohistochemical staining was performed on both groups of tissue samples after treatment. The patients were followed up for 6 months to observe their satisfaction, adverse effects, and scar recurrence in the two groups.

## Histological observation

Normal tissue, hypertrophic scar tissue, and post-treatment tissue from the same patient were fixed with 10% formaldehyde solution, embedded in paraffin, and then sectioned into 5 μm-thick slices, and stored at room temperature. The sections were stained by Hematoxylin-Eosin (HE) staining and then observed under a microscope, the visual fields of vision at a ×100 magnification were randomly selected and photographed.

### Tissue extraction and determination

Under aseptic conditions, hypertrophic scar tissue before and after treatment of the patient was obtained and add appropriate PBS buffer solution, was fully shaken, the precipitation was discarded after centrifugation, and the supernatant was retained for further testing. ELISA assay steps were performed in strict accordance with the reagent specification: collect a tissue sample and prepare a sample extract. Tissue samples are broken with corresponding buffers to release the target protein. The coating agent of the enzyme-labeled plate with a specific antibody was added to the enzyme-labeled plate hole to bind the antibody to the surface of the hole plate. The sample extract is added to the enzyme-labeled plate hole coated with the antibody to bind the target protein to the antibody. Wash the plate with a suitable buffer to remove unbound substances. The secondary antibody that binds specifically to the target protein is added, and the enzyme plate is washed again with buffer to remove the unbound substance. The appropriate substrate is added to catalyze the color reaction of the substrate, and a reaction stopper is added to stop the reaction and prevent further color transformation. The absorbance was read by an enzyme reader to measure the color intensity produced by the reaction. Calculate the concentration of the target protein in the sample.

### Satisfaction evaluation

Satisfaction was evaluated using the self-made questionnaire of the studied hospital. According to the subjective evaluation of patients, it was divided into three levels: satisfaction, basic satisfaction, and dissatisfaction. Satisfaction = satisfaction rate + basic satisfaction rate.

### *Statistical methods*

SPSS 24.0 statistical software was applied to analyze study data. Continuous variables conforming to normal distribution were presented as the mean ± standard deviation (mean ± SD), and an independent sample *t*-test was used for comparison between groups. Categorical data were reported in terms of frequency and percentage and were analyzed with the Chi-Squared test; p < 0.05 was considered significant.

## Results

### *Baseline characteristics*

No obvious differences in sex, age, course of the disease, scar hyperplasia site, Cause of the burn, and the time from burn to treatment were observed between the two groups (p>0.05), as shown in Table 1.

**Table 1:** Clinical characteristics of patients in the two groups.

**Variable**	**Observation group (n = 20)**	**Control group (n = 20)**	***t*/*χ^2^*‐values**	**p‐values**
Sex (male/female)	12/8	10/10	0.404	0.525
Age (years)	35.76 ± 4.27	35.25 ± 4.54	0.366	0.716
Course of disease (months)	9.18 ± 2.17	10.15 ± 2.85	1.211	0.233
Scar hyperplasia site			0.417	0.519
Face	11 (55.00)	13 (65.00)		
Neck	9 (45.00)	7 (35.00)		
Cause of the burn			0.559	0.756
Scalding	11 (55.00)	10 (50.00)		
Thermal	4 (20.00)	6 (30.00)		
Chemical	5 (25.00)	4 (20.00)		
The time from burn to treatment (h)	11.35 ± 2.47	10.87 ± 2.64	0.594	0.557

### *Clinical efficacy assessment*

At 3 months after treatment, VSS scores of the two groups were reduced and the VSS score of the observation group was lower than that of a control group (p < 0.05), seen in Table 2. The overall clinical efficacy of the observation group was higher than that of a control group (p < 0.05), as displayed in Table 3.

**Table 2:** Comparison of VSS score.

**Groups**	**Before treatment**	**After treatment**
Observation group (n = 20)	8.18 ± 1.37	2.29 ± 0.56^a^
Control group (n = 20)	8.26 ± 1.69	4.38 ± 1.15^a^
*t*-values	0.164	7.307
p‐values	0.870	0.000

Note:

a *p* < 0.05 versus before treatment.

**Table 3:** Comparison of Clinical efficacy between two groups.

**Groups**	**Cured**	**Effective**	**Improved**	**Ineffective**	**Total effective rate**
Observation group (n = 20)	5 (25.00)	8 (40.00)	5 (25.00)	2 (10.00)	18 (90.00)
Control group (n = 20)	2 (10.00)	4 (20.00)	5 (25.00)	9 (45.00)	11 (55.00)
*χ^2^*-values					6.144
p‐values					0.013

### *Fibroblast changes in hyperplastic scar tissue*

**Fig. 1:**
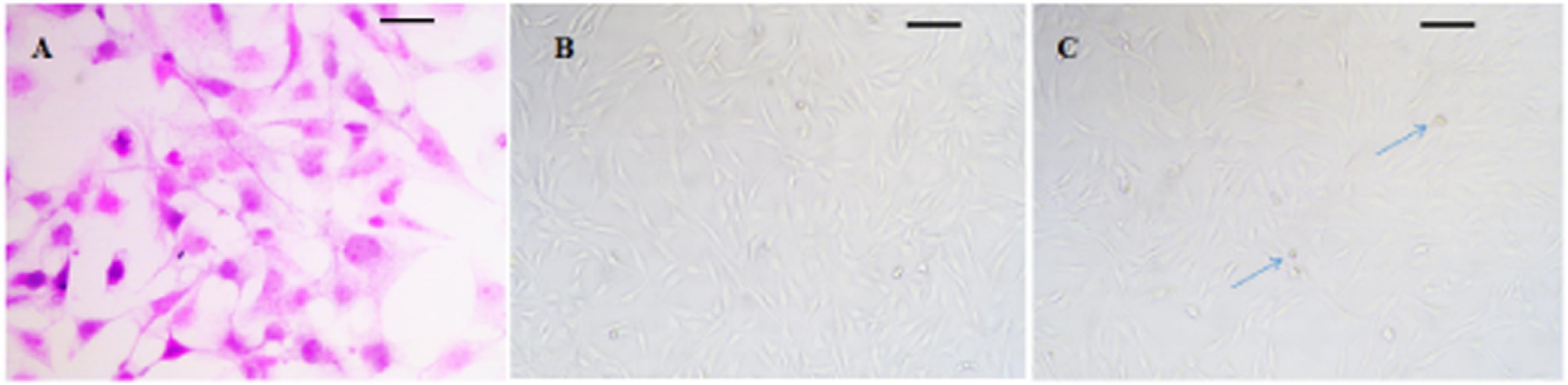
Morphology of fibroblasts in different tissues (ruler shows 250 µm). (A) Normal fibroblasts; (B) Fibroblasts in scar tissue; (C) Fibroblasts after treatment.

HE staining results showed that the fibroblasts were long spindle-shaped or triangular, the nuclei were light blue, and the cytoplasm was pink. After being irradiated by A/A-ES photodynamic therapy, it was observed that the fibroblasts became elliptical or round, the connections between the cells became loose, and the normal arrangement of the cells disappeared. Mitochondrial and rough endoplasmic reticulum ultrastructural changes, are seen from Figure 1.

### *Changes in tissue repair-related cytokine levels*

**Fig. 2:**
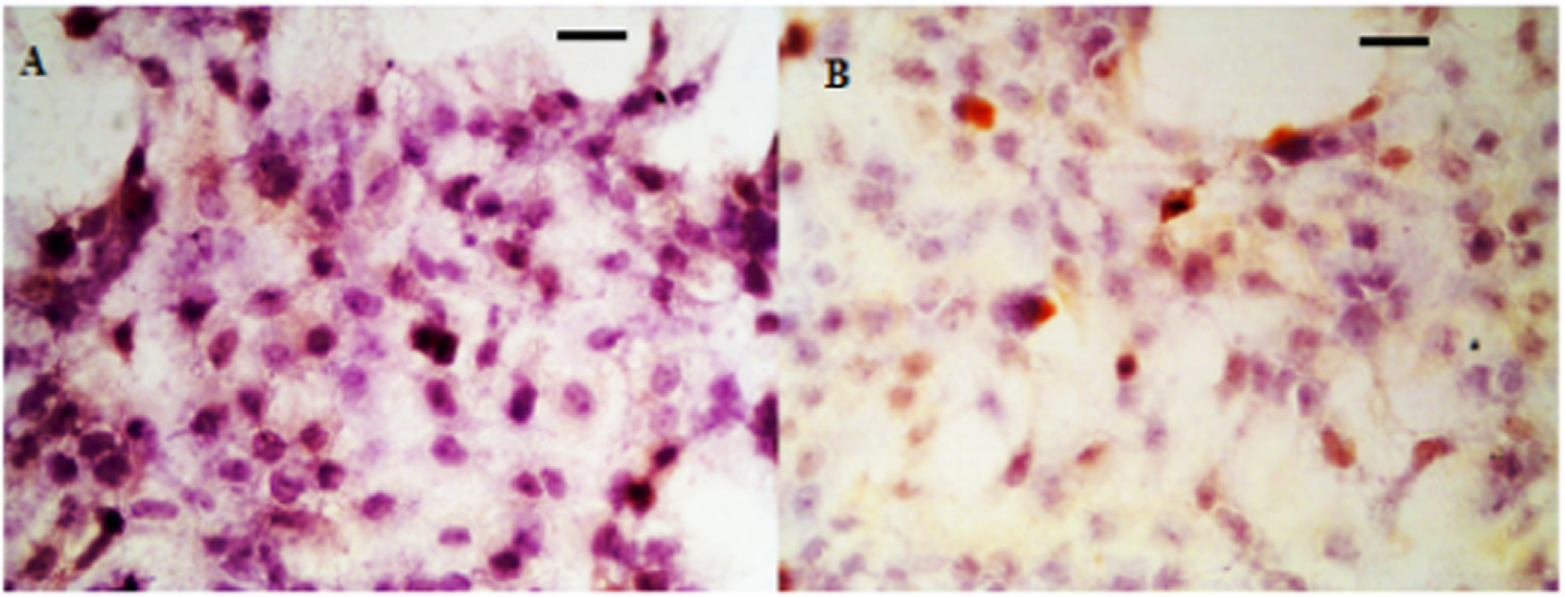
IHC analysis of VEGF in hyperplastic scar tissue. (A) Control group; (B) Observation group after treatment.

**Fig. 3:**
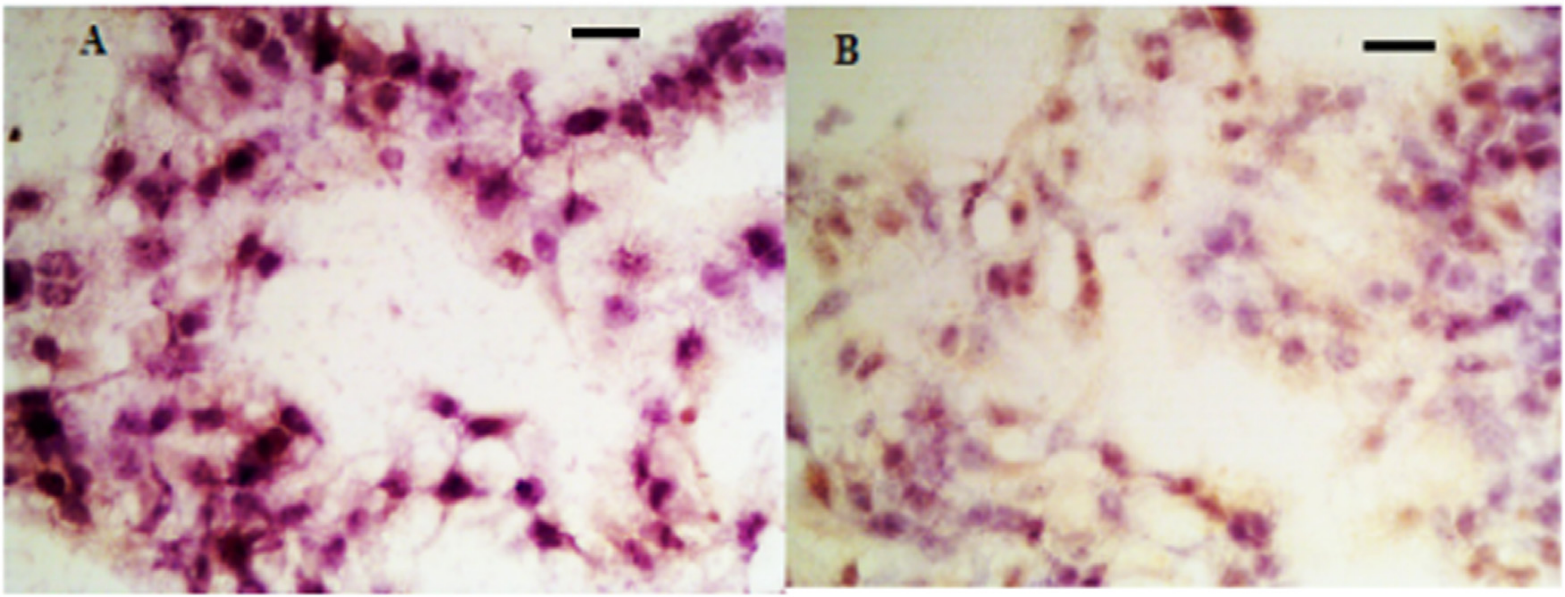
IHC analysis of TGF-β in hyperplastic scar tissue. (A) Control group; (B) Observation group after treatment.

**Fig. 4:**
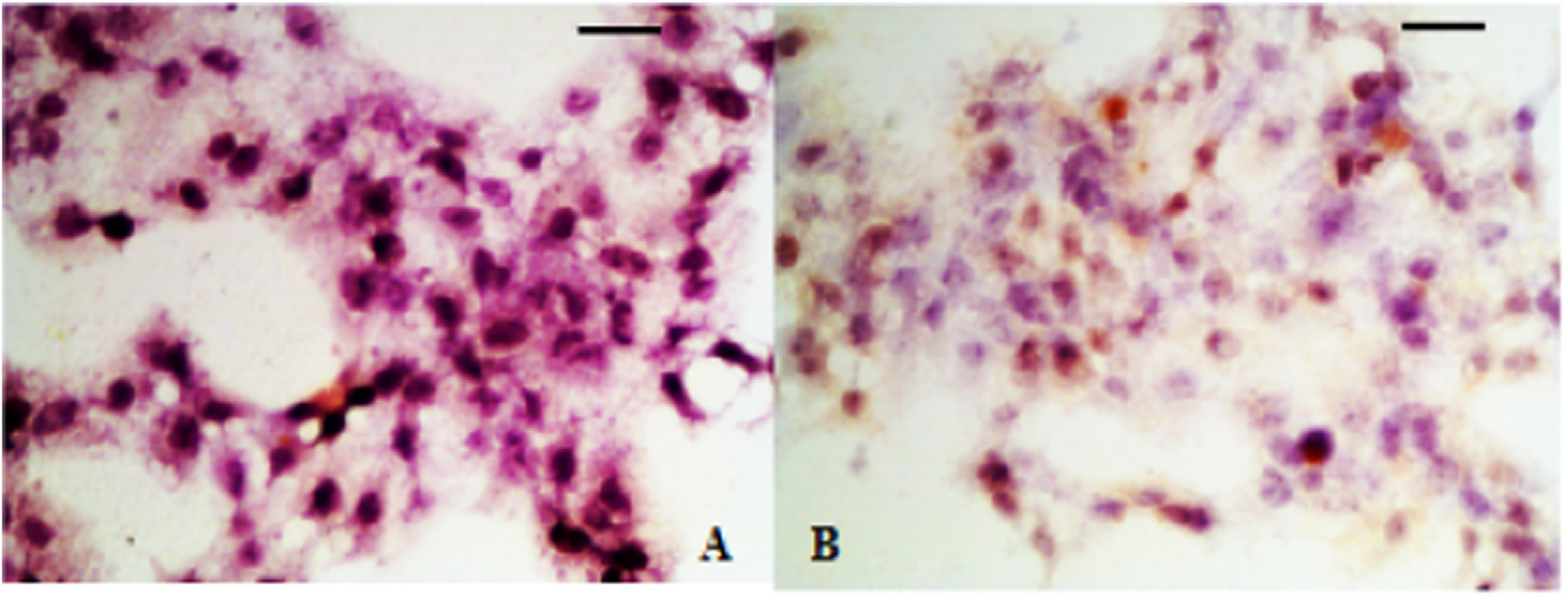
IHC analysis of PDGF in hyperplastic scar tissue. (A) Control group; (B) Observation group after treatment.

Before treatment, the VEGF, TGF-β, PDGF levels did not differ significantly in both groups (p > 0.05). After 3 months of therapy, the VEGF, TGF-β, PDGF levels were distinctly reduced in two groups (p < 0.05), while the VEGF, TGF-β, PDGF levels were lower in the observation group (p < 0.05), as displayed in Table 4. Meanwhile, the immunohistochemistry confirmed the expressions of VEGF, TGF-β, PDGF in the observation group were inhibited more significantly than a control group after treatment, as seen from Figure 2, Fig. 3, Fig. 4.

**Table 4:** Comparison of VEGF, TGF-β, PDGF levels in scar tissue.

**Groups**	**VEGF (μg.mg^−1^)**		**TGF-β (μg.mg^−1^)**		**PDGF (pg.mg^−1^)**	
	**Before treatment**	**After 3 months of treatment**	**Before treatment**	**After 3 months of treatment**	**Before treatment**	**After 3 months of treatment**
Observation group (n = 20)	242.57±35.13	77.42±13.19^a^	84.39±17.12	30.89±6.76^a^	24.65±4.45	10.35±2.71^a^
Control group (n = 20)	251.61±38.21	122.34±20.36^a^	83.27±13.42	51.82±10.18^a^	24.79±3.27	16.48±3.12^a^
*t*-values	0.779	8.281	0.230	7.660	0.113	6.634
p‐values	0.441	0.000	0.819	0.000	0.910	0.000

Note:

a p < 0.05 versus before treatment.

### *Follow-up results*

Six months after treatment, 17 patients in the observation group were satisfied with the aesthetic appearance (85.00%), and 3 patients were dissatisfied (15.00%), with a total satisfaction rate of 85.00%; 11 patients in the control group were satisfied with the aesthetic appearance (55.00%), and 9 patients were dissatisfied (45.00%), with a total satisfaction rate of 55.00%. The observation group was more satisfied with the aesthetic appearance than the control group (p < 0.05), see Table 5. The overall incidence of adverse reactions in the observation group was 10.00%, which was not significantly different from 25.00% in the control group (p > 0.05), as shown in Table 6. Six months after treatment, 6 cases (30.00%) in the control group and 1 case (5.00%) in the observation group relapsed, and the difference was significant between groups (p<0.05).

**Table 5:** Comparison of satisfaction with aesthetics between two groups.

**Groups**	**Satisfaction**	**Dissatisfaction**	**Total satisfaction**
Observation group (n = 20)	17 (80.00)	3(15.00)	17 (85.00)
Control group (n = 20)	11(45.00)	9 (45.00)	11 (55.00)
*χ^2^*-values			4.286
p‐values			0.038

**Table 6:** Comparison of adverse reactions between two groups.

**Groups**	**Folliculitis**	**Atrophy of skin**	**Skin swelling**	**Total incidence**
Observation group (n = 20)	1(5.00)	0 (0.00)	1(5.00)	2 (10.00)
Control group (n = 20)	2 (35.00)	1 (20.00)	2 (45.00)	5 (25.00)
*χ^2^*-values				1.558
p‐values				0.212

## Discussions

Hypertrophic scars can be associated with pain, itching, and sensory allergies, which can affect patients' quality of life.^10^ Photodynamic therapy has the advantages of high selectivity and few side effects, and has shown remarkable effects in the treatment of hypertrophic scar.^11^ The principle of photodynamic therapy for hyperplastic scar is that the photosensitizer first accumulates in hypertrophic scar fibroblasts, and then, under appropriate laser irradiation, the photosensitizer produces ROS, which leads to hypertrophic scar fibroblasts apoptosis.^12^ ALA is currently the best photosensitizer in dermatology local treatment, but its penetration into hypertrophic scar tissue and hypertrophic scar fibroblasts is poor, and the quantum yield of ROS is low, so high-dose ALA or high-intensity laser is usually used clinically. Unfortunately, high doses of ALA can cause damage to the sebaceous glands and epidermis, and high-intensity lasers often cause damage to healthy tissue.^13^ As a result, improving the permeability of ALA and the quantum yield of ROS in the photodynamic treatment of hypertrophic scar has become a research focus.

Currently, ES has attracted a lot of attention as an efficient transdermal drug delivery system for photodynamic therapy of hypertrophic scar. A/A-ES as a functional transdermal system, its a unique structure that can deliver ALA and AuNP together into hypertrophic scar tissue and effectively decompose endogenous hydrogen peroxide in hypertrophic scar tissue to produce oxygen.^14^ The development of A/A-ES opens up a new idea for photodynamic therapy of hypertrophic scar.

Hypertrophic scars are mainly caused by tissue damage and over-repair and are characterized by an increase in fibroblast and collagen matrix. Studies have shown that growth factors play an important role in the process of human cell division, migration, differentiation, and protein expression.^15^ TGF-β is an important growth factor in scar formation, which can promote fibroblast proliferation and increase collagen synthesis;^16^ VEGF is an important regulatory factor for angiogenesis and vascular endothelial cell proliferation, which can promote angiogenesis, tissue repair, and regeneration.^17^ However, a high expression level of VEGF will promote abnormal hypertrophic blood vessels in hypertrophic scar patients to provide nutrients for scar tissue, increase collagen fiber expression and aggravate scar. As an angiogenic factor, PDGF can stimulate the division and proliferation of fibroblasts.^18^ In addition, inflammation plays an important role in the healing process of hypertrophic scars.^19^ At present, many studies have shown that Taohong Siwu Decoction can effectively inhibit the inflammatory response of skin scar tissue and promote scar repair.

In this study, a biocompatible A/A-ES transdermal drug delivery system combined with Taohong Siwu Decoction was applied for the first time in patients with hyperplastic scar after severe burn, and its clinical efficacy and possible mechanism were explored. It was found that the VSS score was lower in the observation group than that in the control group after treatment, and the clinical efficacy was superior to the control group, indicating that the A/A-ES system combined with Taohong Siwu Decoction has a significant clinical effect on patients with hyperplastic scar after severe burn. After HE-staining of scar tissue, it was found that the arrangement of fibroblasts in scar tissue was loose, suggesting that the A/A-ES system combined with Taohong Siwu Decoction may improve hyperplastic scar by inhibiting the proliferation of fibroblasts. A recent study found that ALA-ES can improve hyperplastic scar by promoting hypertrophic scar fibroblast apoptosis, which obtained similar conclusions to this study.^20^ In Tan's study,^21^ the Taohong Siwu Decoction has been proven to inhibit fibrosis proliferation through the TGFBR1 signaling pathway. In order to further explore the mechanism of treatment, this study further analyzed the changes in growth factor levels before and after treatment. The results showed that the levels of VEGF, TGF-β, and PDGF in the observation group were lower than those in the control group after treatment, which further revealed that the A/A-ES system combined with Taohong Siwu Decoction may improve hyperplastic scar by reducing the cytokines related to tissue repair. After exposure to light, the photochemical reaction mediated by protoporphyrin IX will produce a large number of ROSs to destroy the rough endoplasmic reticulum, mitochondria and ribosomes, leading to the destruction of organelles. The damaged organelles will lead to protein synthesis obstacles and energy production restrictions, thus affecting the proliferation of fibroblasts.^22^ Modified photodynamic therapy has good permeability to hypertrophic scar tissue and high ROS quantum yield, so its clinical effect on the hypertrophic scar is superior to that of conventional laser therapy.

The results further showed that 6-months after treatment, patients in the observation group were more satisfied than those in the control group, there was no significant difference in the occurrence of adverse reactions between the two groups, and the recurrence rate in the observation group was lower than that in control group, indicating that modified photodynamic therapy combined with Taohong Siwu Decoction in treating hyperplastic scar has higher safety and satisfaction, and can reduce the recurrence rate. This study provides an effective method for the clinical treatment of hyperplastic scars after severe burns. However, there are some limitations in this study. Firstly, this research is a prospective study with a limited sample size and single source. Therefore, it is necessary to carry out a large number of prospective researches to confirm the conclusion. Secondly, the research on the mechanism of improved photodynamic therapy is not deep enough.

## Conclusions

Modified photodynamic therapy combined with Taohong Siwu Decoction has a remarkable effect on hyperplastic scars after severe burn, which can effectively improve the color, texture, and thickness of scars in patients. It can inhibit the proliferation of fibroblasts in hypertrophic scar and improve the level of cell growth factor. Additionally, the therapy obtained higher aesthetic satisfaction and safety, and the recurrence rate was significantly reduced, which is worthy of clinical application.

## Ethics approval and consent to participate

The ethics approval was reviewed and approved by the Ethic Committee of Lishui People's Hospital and informed written consent from all of the patients. All methods were carried out in accordance with the STROBE Statement.

## Consent for publishing

All of the authors have Consented to publish this research.

## Availability of data and materials

The data are free to access and available upon request.

## Authors' contributions

Each author has made an important scientific contribution to the study and has assisted with the drafting or revising of the manuscript.

## Declaration of Competing Interest

The authors declare no conflicts of interest.
